# Excessive sweating in a male patient caused by milnacipran

**DOI:** 10.4103/0019-5545.49465

**Published:** 2009

**Authors:** Yutaka Shinohara

**Affiliations:** Department of Psychiatry, Tokai University Hachioji Hospital, 1838, Ishikawa-machi, Hachioji, Tokyo 192-0032, Japan. E-mail: y-shino@umin.net

Sir,

It is known that SSRIs causes sweating. We report on a man who developed excessive sweating caused by milnacipran, an SNRI used usually in Japan.

This report describes a 54-year old male who visited a psychiatric outpatient service in August X. The patient worked at an electric equipment manufacturing plant as a circuit design engineer. He began feeling weary of going to work since he had been assigned to a different position within the company two weeks prior to his visit. His description of symptoms at the outpatient visit included loss of appetite, arousal during sleep, significant fatigue in the morning, depression, and loss of interest in hobbies. He did not report any problems with either his personal or family life and did not have any significant health history. There was no previous record of drug abuse or alcoholism and no indication of previous hyperthymia. Moreover, the results of a blood test and a brain CT scan were normal. Based on these findings, the patient was diagnosed with a single major depressive episode.

Medication was initiated with oral administration of milnacipran (50 mg/day), which was increased a week later to 100 mg/day. The patient's depression improved after four weeks, however he continued to experience morning fatigue and arousal during sleep. These problems also improved after five weeks, but the patient continued to experience anxiety when going out to the point of having sweaty palms. The blood level of free T3, free T4 and thyroid stimulating hormone was normal. This symptom progressed into the sixth week and a small amount of exercise resulted in excessive sweating of the palms and feet. Treatment with milnacipran was consequently changed to oral administration of maprotiline (60 mg/day), which was increased a week later to 80 mg/day. The patient was stable for the next six months with no abnormal sweating.

Previous reports have indicated that selective serotonin reuptake inhibitors (SSRIs) may cause sweating, 1) while a limited number of cases have reported on the same side effect caused by milnacipran. 2) Since sweating may cause difficulties in continuing treatment with milnacipran, it is important to observe this particular side effect of milnacipran in future studies.
